# Abdominal aortic calcification index and prognosis in patients with chronic kidney disease: a meta-analysis

**DOI:** 10.3389/fmed.2026.1907104

**Published:** 2026-09-09

**Authors:** Yicong Pan, Wenhong Jiang, Ming Hu, Xiao Qin

**Affiliations:** Department of Vascular Surgery, The First Affiliated Hospital of Guangxi Medical University, Nanning, China

**Keywords:** aortic calcification index, cardiovascular events, chronic kidney disease, meta-analysis, survival

## Abstract

**Background:**

Abdominal aortic calcification index (ACI), a quantitative computed tomography–based measure of abdominal aortic calcification burden, has been increasingly investigated as a prognostic marker in patients with chronic kidney disease (CKD). However, the association between ACI and clinical outcomes in CKD remains uncertain. This meta-analysis evaluated the association of baseline ACI with cardiovascular event-free survival (CVEFS) and overall survival (OS) in patients with CKD.

**Methods:**

PubMed, Embase, Web of Science, Wanfang Data, and CNKI were searched for cohort studies evaluating the association between baseline ACI and prognosis in adult patients with CKD. Hazard ratios (HRs) comparing high versus low ACI were pooled using random-effects models accounting for the influence of potential heterogeneity. Prespecified subgroup, sensitivity, and meta-regression analyses were performed.

**Results:**

Eleven cohort studies involving 1,914 patients with CKD were included. Nine studies reported CVEFS and five reported OS. Pooled analysis showed that a high ACI was associated with poorer CVEFS (HR = 3.20, 95% CI: 2.21–4.64; I^2^ = 44%) and OS (HR = 2.69, 95% CI: 1.86–3.89; I^2^ = 0%). The association with CVEFS remained significant across study design and renal replacement therapy subgroups. Stronger associations with CVEFS were observed in studies with a mean age < 60 years as compared to ≥ 60 years (HR = 5.46 vs. 2.27; *p* for subgroup difference = 0.01) and ACI cutoff values ≥ 30% compared to < 30% (HR = 8.53 vs. 2.37; *p* < 0.001). Meta-regression identified ACI cutoff value as the only significant study characteristic associated with the strength of the association between ACI and CVEFS (coefficient = 0.033, *p* = 0.02).

**Conclusion:**

A high baseline ACI was associated with poorer CVEFS and OS in patients with CKD. ACI may have potential as an imaging biomarker for prognostic risk stratification in this population, although these findings should be interpreted cautiously and confirmed in future prospective studies.

**Systematic review registration:**

https://www.crd.york.ac.uk/prospero/, identifier CRD420261421928.

## Introduction

Chronic kidney disease (CKD) is a major global public health problem affecting approximately 10–15% of the adult population worldwide (1, 2). Despite advances in medical management and renal replacement therapies, patients with CKD continue to experience substantially increased risks of cardiovascular morbidity and mortality compared with the general population (3). The progression of CKD is frequently accompanied by a broad spectrum of metabolic, inflammatory, and vascular abnormalities that contribute to adverse clinical outcomes (4, 5). Notably, cardiovascular disease represents the leading cause of death among patients with CKD, accounting for a substantial proportion of excess mortality across all stages of the disease (6). Given the heterogeneous clinical course of CKD, accurate risk stratification is essential for identifying high-risk patients, optimizing monitoring strategies, and guiding preventive interventions (7). Consequently, considerable efforts have been devoted to identifying reliable prognostic markers that can improve outcome prediction beyond traditional cardiovascular risk factors (7).

Vascular calcification is one of the most prevalent and clinically important cardiovascular complications of CKD (8). Disturbances in mineral and bone metabolism, chronic inflammation, oxidative stress, and accelerated vascular aging promote the development and progression of vascular calcification in this population (9, 10). Among the various forms of vascular calcification, abdominal aortic calcification (AAC) has attracted particular interest because it reflects systemic atherosclerotic and arteriosclerotic burden and is readily detectable using imaging techniques (11–13). Progressive calcification of the abdominal aorta may contribute to increased arterial stiffness, elevated pulse pressure, impaired coronary perfusion, left ventricular remodeling, and reduced vascular compliance, thereby increasing the risks of cardiovascular events and death (14). Previous studies have consistently suggested that the presence and severity of AAC are associated with adverse cardiovascular outcomes in patients with CKD, although the magnitude of these associations has varied across studies (15, 16).

The abdominal aortic calcification index (ACI) is a quantitative computed tomography (CT)-based measure that estimates the percentage of the abdominal aortic wall affected by calcification (17, 18). In a typical assessment protocol, consecutive CT slices of the abdominal aorta are divided into multiple circumferential segments, and the proportion of calcified segments is used to calculate the ACI as a percentage, providing a continuous measure of calcification burden (17, 18). Compared with semiquantitative radiographic scoring systems and simple assessments of the presence or absence of calcification, ACI offers greater objectivity, reproducibility, and sensitivity for quantifying calcification severity. In recent years, an increasing number of cohort studies have investigated the prognostic significance of ACI in patients with predialysis CKD, dialysis-dependent kidney disease, and kidney transplant recipients (19–29). However, the reported associations between ACI and clinical outcomes have not been entirely consistent, and no meta-analysis has comprehensively synthesized the available evidence. Therefore, the present study aimed to systematically evaluate the associations between baseline ACI and cardiovascular event-free survival (CVEFS) and overall survival (OS) in patients with CKD through a meta-analysis of cohort studies.

## Methods

This meta-analysis was conducted in accordance with recognized standards for systematic reviews and meta-analyses. The methodology adhered to the recommendations of the PRISMA 2020 guideline (30) and the Cochrane Handbook for Systematic Reviews of Interventions (31), covering all stages from protocol development and literature screening to data extraction, quantitative synthesis, and interpretation of findings. The review protocol was prospectively registered in PROSPERO (CRD420261421928).

### Database search

A comprehensive search of the literature was performed in PubMed, Embase, Web of Science, Wanfang Data, and China National Knowledge Infrastructure (CNKI) to identify all relevant studies. Search terms were developed around three key concepts: aortic calcification index, chronic kidney disease, and clinical outcomes, with (“aortic calcification index” OR “Aortic Calcification Index” OR ACI OR “abdominal aortic calcification index” OR “abdominal aortic calcification percentage” OR “aortic calcification percentage”) AND (“renal” OR “kidney” OR “hemodialysis” OR “dialysis”) AND (“death” OR “mortality” OR “survival” OR “events” OR “outcome” OR “cardiovascular” OR “prognosis”). Synonyms, abbreviations, and related expressions for each concept were combined using Boolean operators to maximize retrieval. Eligibility was restricted to peer-reviewed full-text studies involving human participants and published in either English or Chinese. To ensure completeness, the reference lists of relevant reviews and included articles were also screened manually for additional records. Database searches covered the period from database inception to April 12, 2026. The complete search strategies used for each database are provided in Supplementary File 1.

### Study inclusion and exclusion criteria

The selection of studies was guided by the PICOS principle:

Population (P): Adult patients ( ≥ 18 years) with CKD were included, comprising predialysis CKD, maintenance dialysis (hemodialysis or peritoneal dialysis), and kidney transplant recipients. Studies enrolling mixed populations were included if data for patients with CKD could be extracted separately.

Exposure (I/E): The exposure of interest was the abdominal ACI measured using CT at baseline. Studies reporting outcomes according to ACI categories (e.g., high versus low ACI) were considered eligible.

Comparator (C): Comparisons were made between patients with higher and lower baseline ACI levels, as defined by the individual studies.

Outcomes (O): The primary outcome was CVEFS, and the secondary outcome was OS, compared between patients with CKD and a high versus low abdominal ACI at baseline. CVEFS was generally defined as the time from baseline ACI assessment to the occurrence of a cardiovascular event, including major adverse cardiovascular events, cardiovascular hospitalization, myocardial infarction, stroke, heart failure, cardiovascular death, or composite cardiovascular outcomes, according to the definitions used in the individual studies. OS was generally defined as the time from baseline ACI assessment to death from any cause during follow-up.

Study Design (S): Prospective and retrospective cohort studies were included.

Studies were excluded if they (1) enrolled pediatric populations, animals, or participants without CKD; (2) did not assess abdominal ACI; (3) evaluated other vascular calcification measures without reporting ACI separately; (4) lacked outcomes of CVEFS or OS; or (5) provided insufficient data to estimate effect sizes. Cross-sectional studies, case-control studies, case reports, case series, reviews, editorials, letters, and duplicate publications were also excluded. When multiple reports originated from the same cohort, the study with the largest sample size was retained.

### Study quality assessment

All stages of the review process were conducted independently by two investigators, including study identification, eligibility assessment, data extraction, and quality appraisal. Discrepancies were resolved through discussion, with adjudication by a third reviewer when consensus could not be achieved. Study quality was assessed using the Newcastle–Ottawa Scale (NOS) (32), which evaluates the risk of bias in observational studies across participant selection, group comparability, and outcome ascertainment. Scores range from 0 to 9, with a score of 8 or higher indicating high methodological quality.

### Data collection

Two reviewers independently extracted study data using a standardized form developed before data collection. Extracted information included study-level characteristics (author, publication year, country, and design), demographic and clinical characteristics of the patients (diagnosis, renal replacement therapy [RRT] status, sample size, age, sex, and diabetic status), protocol of ACI evaluation, methods to determine the cutoffs of ACI, cutoff values of ACI in each study, outcomes reported, and factors adjusted in the analysis of the association between ACI and outcomes of patients with CKD.

### Statistical analyses

The associations between abdominal ACI and CVEFS and OS in patients with CKD were quantified by pooling hazard ratios (HRs) and corresponding 95% confidence intervals (CIs) comparing patients with high versus low baseline ACI (31). Before quantitative synthesis, all effect estimates were reviewed and harmonized to ensure a consistent direction of effect, such that HRs > 1 uniformly represented a higher hazard of cardiovascular events (corresponding to poorer cardiovascular event-free survival) or all-cause death in patients with high ACI. When multiple adjusted models were reported, the estimates derived from the model with the most comprehensive adjustment were selected for analysis. If required, effect sizes and standard errors were calculated from the available 95% CIs or p values. Prior to data synthesis, all effect estimates were transformed to the natural logarithmic scale to improve distribution normality and stabilize variances (31). Between-study inconsistency was examined using Cochran’s Q statistic and quantified with the I^2^ metric (33). Heterogeneity was interpreted as low, moderate, or substantial when I^2^ values were < 25%, 25–75%, and > 75%, respectively. Given the anticipated clinical and methodological diversity among studies, pooled estimates were generated using an inverse-variance random-effects model based on the DerSimonian–Laird method (31). In addition, sensitivity analyses were performed using the restricted maximum likelihood (REML) random-effects model to further evaluate the robustness of the pooled estimates. The robustness of the findings was investigated through leave-one-out sensitivity analyses, whereby the meta-analysis was repeated after sequential removal of each individual study (34). For the primary outcome of CVEFS, prespecified subgroup analyses were conducted according to study design (prospective vs. retrospective), RRT status (pre-dialysis CKD, on dialysis, or kidney transplant recipients), mean ages of the patients, proportions of men, cutoffs of ACI, mean follow-up durations, analytic models (univariate vs. multivariate), and NOS scores. For subgroup analyses according to ACI, a cutoff value of 30% was prespecified because it approximated the upper range of thresholds reported in the included studies and allowed exploration of whether studies using relatively higher versus lower ACI thresholds yielded different effect estimates. For all other study-level variables, median values were used as subgroup cutoffs to achieve a balanced distribution of studies between subgroups. Moreover, univariate meta-regression analyses were conducted to assess whether study characteristics influenced the association between ACI and CVEFS in patients with CKD (31). Covariates examined included sample size, mean age, proportion of men, proportion of diabetic patients, ACI cutoff value, mean follow-up duration, and NOS quality score. Potential small-study effects and publication bias were explored by visual assessment of funnel plots and formally tested using Egger’s linear regression method (35). Statistical significance was determined using a two-tailed p value of less than 0.05. All quantitative analyses were carried out using RevMan version 5.3 (Cochrane Collaboration, Oxford, UK) and Stata version 17.0 (StataCorp, College Station, TX, USA).

## Results

### Database search results

**FIGURE 1:**
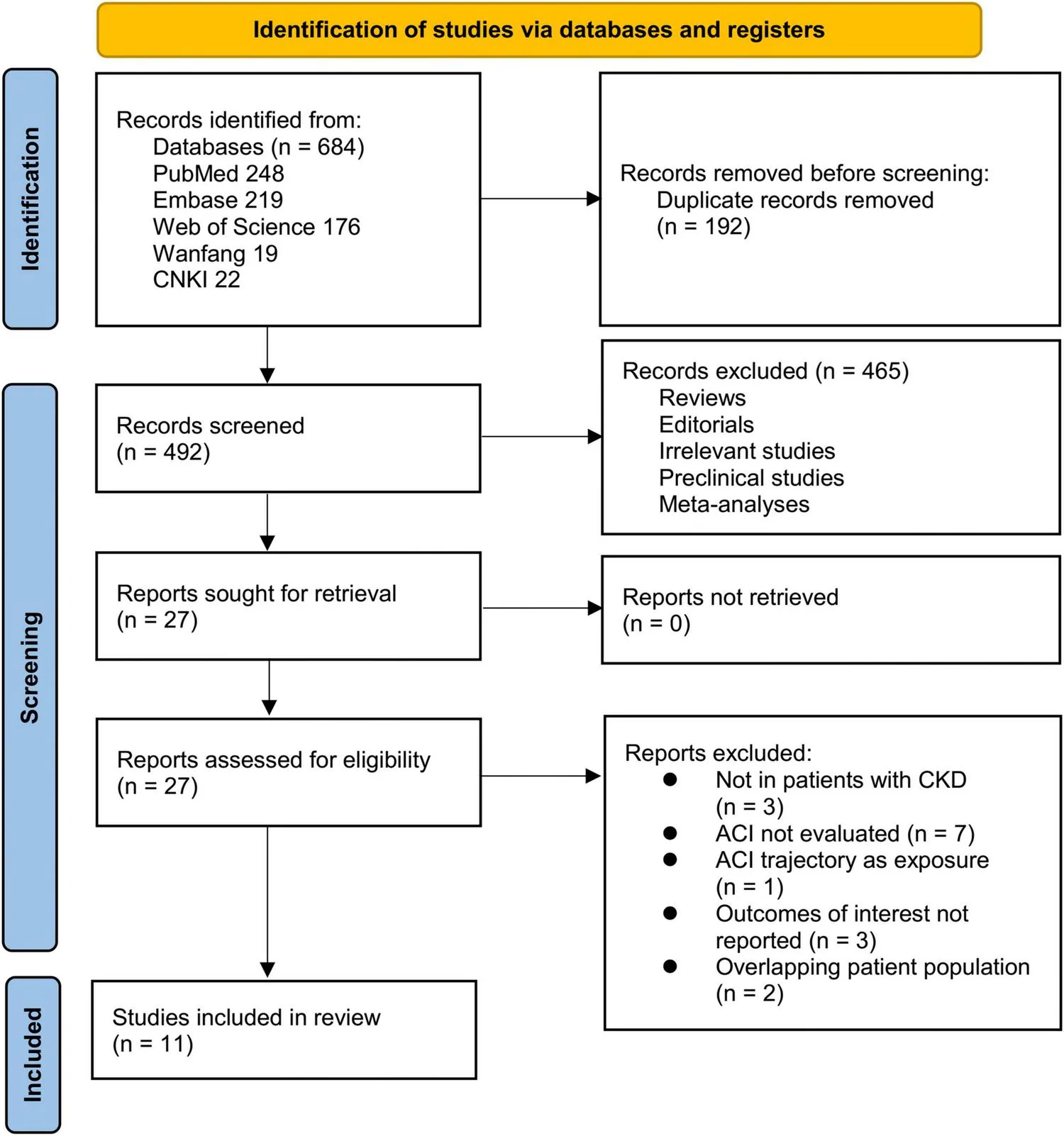
Flow diagram of the study selection process.

Figure 1 summarizes the study identification and selection process. The database search yielded 684 potentially relevant records, of which 192 were duplicates and subsequently removed. Screening of the remaining citations resulted in the exclusion of 465 records at the title and abstract stage. Twenty-seven articles were retrieved for detailed full-text assessment, and 16 were excluded after eligibility evaluation. Ultimately, 11 studies satisfied the inclusion criteria and were included in the meta-analysis (19–29).

### Overview of study characteristics

The main characteristics of the included studies are summarized in Table 1. A total of 11 cohort studies published between 2005 and 2026 were included, comprising 1,914 patients with CKD. Of these, seven studies adopted a prospective cohort design (19–21, 24, 25, 28, 29) and four adopted a retrospective cohort design (22, 23, 26, 27). The studies were conducted predominantly in Asia, including Japan (19–21, 24, 26), Korea (22, 23, 27), China (28, 29), with one additional study conducted in Egypt (25), indicating relatively limited geographic diversity. The mean age of the study populations ranged from 42.7 to 67.4 years, the proportion of men ranged from 41.4 to 69.7%, and the prevalence of diabetes mellitus ranged from 10.0 to 67.1%. The included populations represented a broad spectrum of CKD, including patients receiving hemodialysis and/or peritoneal dialysis (19, 20, 22, 23, 27), predialysis CKD (21, 24, 29), and kidney transplant recipients (26). Abdominal ACI was assessed using non-contrast abdominal CT in all studies, although the anatomical range, slice thickness, and methods used to define ACI cutoffs varied. Three studies used predefined ACI thresholds (19, 21, 29), five used median values (20, 22, 23, 25, 27), two used receiver operating characteristic (ROC) curve-derived cutoffs (26, 28), and one study defined the cutoff according to tertile distribution (24). Accordingly, the ACI cutoff values ranged from 12.3 to 64.0%. Median follow-up durations ranged from 1.4 to 6.8 years. The outcome of CVEFS was reported in nine studies (20–27, 29), and the outcome of OS was reported in five studies (19, 20, 24, 25, 28). The definitions/components of cardiovascular events reported in the individual studies evaluating CVEFS are summarized in Supplementary Table 1. Seven studies (20–24, 27, 28) adjusted for important confounding variables, commonly including age, sex, diabetes, cardiovascular disease, renal function, nutritional parameters, and cardiac function measures, to a varying degree, whereas four studies (19, 25, 26, 29) reported unadjusted analyses only.

**TABLE 1:** Characteristics of the included studies.

Study	Country	Design	Patient characteristics	No. of patients	Mean age (years)	Men (%)	DM (%)	Protocol of ACI evaluation	Methods for defining the cutoff of ACI	Cutoff values of ACI (%)	Median follow-up duration (years)	Outcomes reported	Variables adjusted
Kushiya et al. (19)	Japan	PC	Patients receiving maintenance HD	84	64.8	45.2	22.0	Non-contrast abdominal CT, abdominal aorta, above the bifurcation of the common iliac artery, 10 scans at 1-cm intervals, 120 segments in total	Predefined	30.0	2.0	OS	None
Nishiura et al. (20)	Japan	PC	New HD patients	99	58.9	41.4	41.4	Non-contrast abdominal CT, abdominal aorta, anatomical range and number of slices not specified	Median	30.5	3.5	CVEFS and OS	Age, sex, albumin, and previous CVD
Hanada et al. (21)	Japan	PC	Pre-dialysis CKD patients stages 3–5	101	66.6	63.4	29.7	Non-contrast abdominal CT, abdominal aorta, above the bifurcation of the common iliac artery, 10 scans at 1-cm intervals, 120 segments in total	Predefined	20.0	4.0	CVEFS	Age, sex, serum albumin, eGFR, and DM
Yoon et al. (22)	Korea	RC	Patients receiving maintenance HD	128	64.0	55.5	67.1	Non-contrast abdominal CT, abdominal aorta, anatomical range and number of slices not specified	Median	21.4	1.4	CVEFS	Age, sex, dialysis vintage, DM, CCI, BMI, LVEF, LVSV, E/A ratio, and E/E’ ratio
Yoon et al. (23)	Korea	RC	Patients receiving continuous ambulatory PD	92	55.0	52.2	54.3	Non-contrast abdominal CT, abdominal aorta, anatomical range and number of slices not specified	Median	18.9	2.9	CVEFS	Age, sex, DM, previous CVD, hemoglobin, albumin, CRP, LAD, and LVEF
Tatami et al. (24)	Japan	PC	Asymptomatic CKD patients without dialysis	347	67.4	69.7	34.9	Non-contrast abdominal CT, abdominal aorta, from takeoff of renal artery to bifurcation into common iliac arteries, number of slices not specified	T3:T1^a^	23.5	3.5	CVEFS and OS	Age, sex, BMI, current smoking, SBP, DM, dyslipidemia, phosphorus, corrected calcium, intact PTH, hemoglobin, and albumin
NasrAllah et al. (25)	Egypt	PC	Patients receiving maintenance HD	93	42.7	48.0	10.0	Non-contrast abdominal CT, abdominal aorta, just above aortic bifurcation, 10 cuts at 1-cm intervals ascending, 120 segments in total	Median	12.3	3.9	CVEFS and OS	None
Mursawa et al. (26)	Japan	RC	Adult kidney transplant recipients	100	45.0	62.0	10.0	Non-contrast abdominal CT, abdominal aorta, above bifurcation of common iliac artery, 10 scans at 0.5-cm intervals ascending, 120 segments in total	ROC curve analysis derived cutoff	64.0	4.3	CVEFS	None
Tsai et al. (28)	Taiwan (China)	PC	Patients undergoing PD	123	54.1	43.0	20.0	Non-contrast abdominal CT, abdominal aorta, just above aortic bifurcation, number of slices not specified	ROC curve analysis derived cutoff	39.0	6.8	OS	Age, sex, DM, HTN, LVEF
Kim et al. (27)	Korea	RC	Patients receiving maintenance HD or PD	587	60.6	54.9	56.6	Non-contrast abdominal CT, abdominal aorta, anatomical range and number of slices not specified	Median	17.2	3.1	CVEFS	Age, sex, DM, history of CVD, eGFR, hemoglobin, albumin, AST, hs-CRP, and use of non-calcium-based phosphate binders
Liu et al. (29)	China	PC	Pre-dialysis CKD patients stage 5	160	51.7	51.0	40.0	Non-contrast abdominal CT, abdominal aorta, anatomical range and number of slices not specified	Predefined	30.0	2.0	CVEFS	None

ACI, abdominal aortic calcification index; AST, aspartate aminotransferase; BMI, body mass index; CCI, Charlson comorbidity index; CKD, chronic kidney disease; CRP, C-reactive protein; CT, computed tomography; CVD, cardiovascular disease; CVEFS, cardiovascular event-free survival; DM, diabetes mellitus; E/A ratio, ratio of early (E) to late (A) transmitral flow velocity; E/E’ ratio, ratio of early transmitral flow velocity to early diastolic mitral annular velocity; eGFR, estimated glomerular filtration rate; HD, hemodialysis; hs-CRP, high-sensitivity C-reactive protein; HTN, hypertension; LAD, left atrial dimension; LVEF, left ventricular ejection fraction; LVSV, left ventricular stroke volume; NOS, Newcastle–Ottawa Scale; OS, overall survival; PC, prospective cohort; PD, peritoneal dialysis; PTH, parathyroid hormone; RC, retrospective cohort; ROC, receiver operating characteristic; SBP, systolic blood pressure; T3:T1, ratio of the third to the first tertile.

*^a^*T3:T1 indicates the comparison of the highest tertile (T3) versus the lowest tertile (T1) of ACI, from which the hazard ratio was extracted for the high-versus-low ACI analysis.

### Study quality evaluation

The methodological quality of the included studies was assessed using the NOS, and the results are presented in Table 2. NOS scores ranged from 6 to 9 points, with a median score of 7. Three studies (21, 24, 28) achieved the maximum score of 9, reflecting excellent methodological quality with adequate cohort selection, adjustment for important confounders, outcome assessment, and follow-up. Two studies (20, 27) scored 8, indicating generally high quality with only minor methodological limitations. Another three (22, 23, 25) studies scored 7, while the remaining three studies (19, 26, 29) scored 6, mainly because of limited adjustment for confounding factors, concerns regarding cohort representativeness, or insufficient follow-up duration. Overall, ascertainment of exposure and outcome assessment was adequate across all studies, and all cohorts were confirmed to be free of the outcome of interest at baseline. Most studies controlled for age, sex, and additional confounding variables, although adjustment strategies varied considerably between studies. Despite some limitations in representativeness and confounder adjustment in a subset of studies, the overall methodological quality was considered moderate to high, supporting the reliability of the pooled estimates. Nevertheless, differences in study populations, ACI cutoff definitions, and adjustment models may have contributed to residual heterogeneity among the included studies.

**TABLE 2:** Study quality evaluation via the Newcastle-Ottawa Scale.

Study	Representativeness of the exposed cohort	Selection of the non-exposed cohort	Ascertainment of exposure	Outcome not present at baseline	Control for age and sex	Control for other confounding factors	Assessment of outcome	Enough long follow-up duration	Adequacy of follow-up of cohorts	Total
Kushiya et al. (19)	1	1	1	1	0	0	1	0	1	6
Nishiura et al. (20)	1	1	1	1	1	1	1	0	1	8
Hanada et al. (21)	1	1	1	1	1	1	1	1	1	9
Yoon et al. (22)	0	1	1	1	1	1	1	0	1	7
Yoon et al. (23)	0	1	1	1	1	1	1	0	1	7
Tatami et al. (24)	1	1	1	1	1	1	1	1	1	9
NasrAllah et al. (25)	1	1	1	1	0	0	1	1	1	7
Mursawa et al. (26)	0	1	1	1	0	0	1	1	1	6
Tsai et al. (28)	1	1	1	1	1	1	1	1	1	9
Kim et al. (27)	0	1	1	1	1	1	1	1	1	8
Liu et al. (29)	1	1	1	1	0	0	1	0	1	6

### Association between ACI and outcome of patients with CKD

**FIGURE 2:**
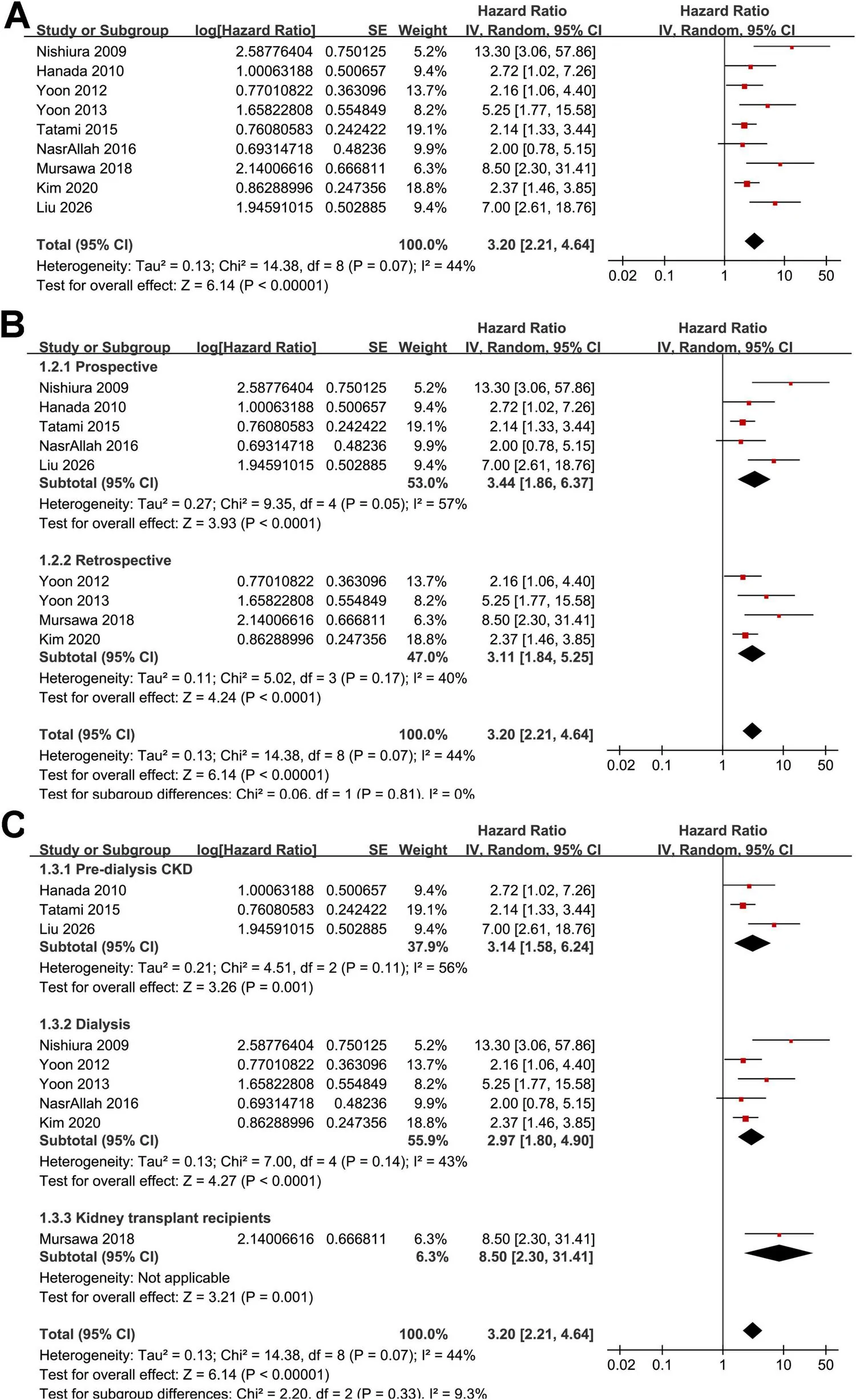
Forest plots showing the meta-analysis of the association between ACI and CVEFS of patients with CKD: **(A)** forest plots for the overall meta-analysis; **(B)** forest plots for the subgroup analysis according to study design; and **(C)** forest plots for the subgroup analysis according to renal replacement therapy status.

The pooled analysis of the 9 studies (20–27, 29) showed that a high ACI at baseline was associated with poor CVEFS in patients with CKD (HR: 3.20, 95% CI: 2.21 to 4.64, *p* < 0.001; Figure 2A) with moderate heterogeneity (*p* for Cochrane Q test = 0.07; I^2^ = 44%). Additional sensitivity analysis using the REML random-effects model yielded an almost identical pooled estimate (HR: 3.18, 95% CI: 2.21 to 4.59; Supplementary Figure 1A), further supporting the stability of the findings. Leave-one-out sensitivity analyses showed similar results, with HR ranging from 2.85 to 3.58 (all *p* < 0.05), indicating the robustness of the overall estimate.

**FIGURE 3:**
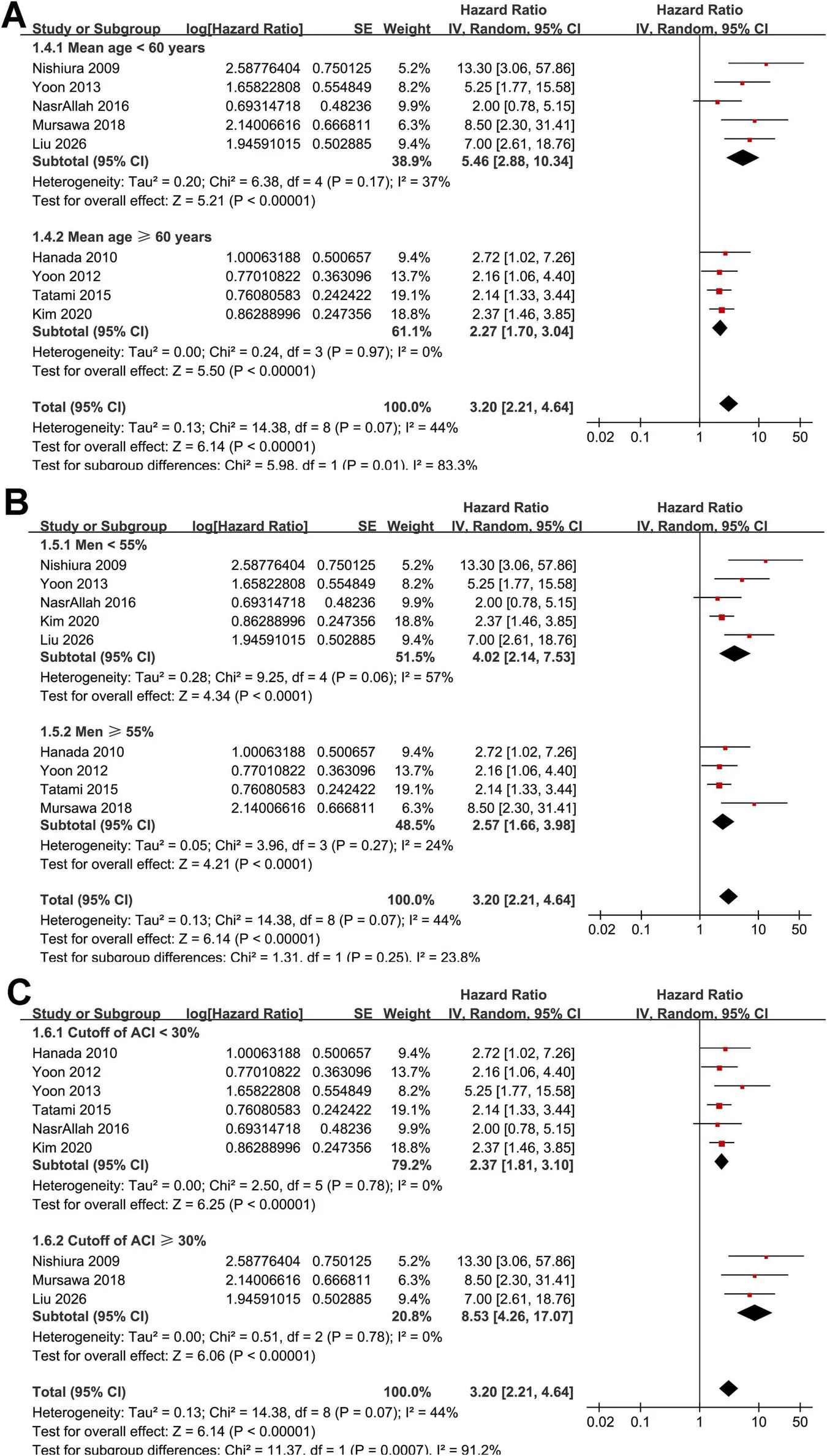
Forest plots showing the subgroup analyses of the association between ACI and CVEFS of patients with CKD: **(A)** forest plots for the subgroup analysis according to mean ages of the patients; **(B)** forest plots for the subgroup analysis according to proportions of men; and **(C)** forest plots for the subgroup analysis according to cutoffs of ACI.

**FIGURE 4:**
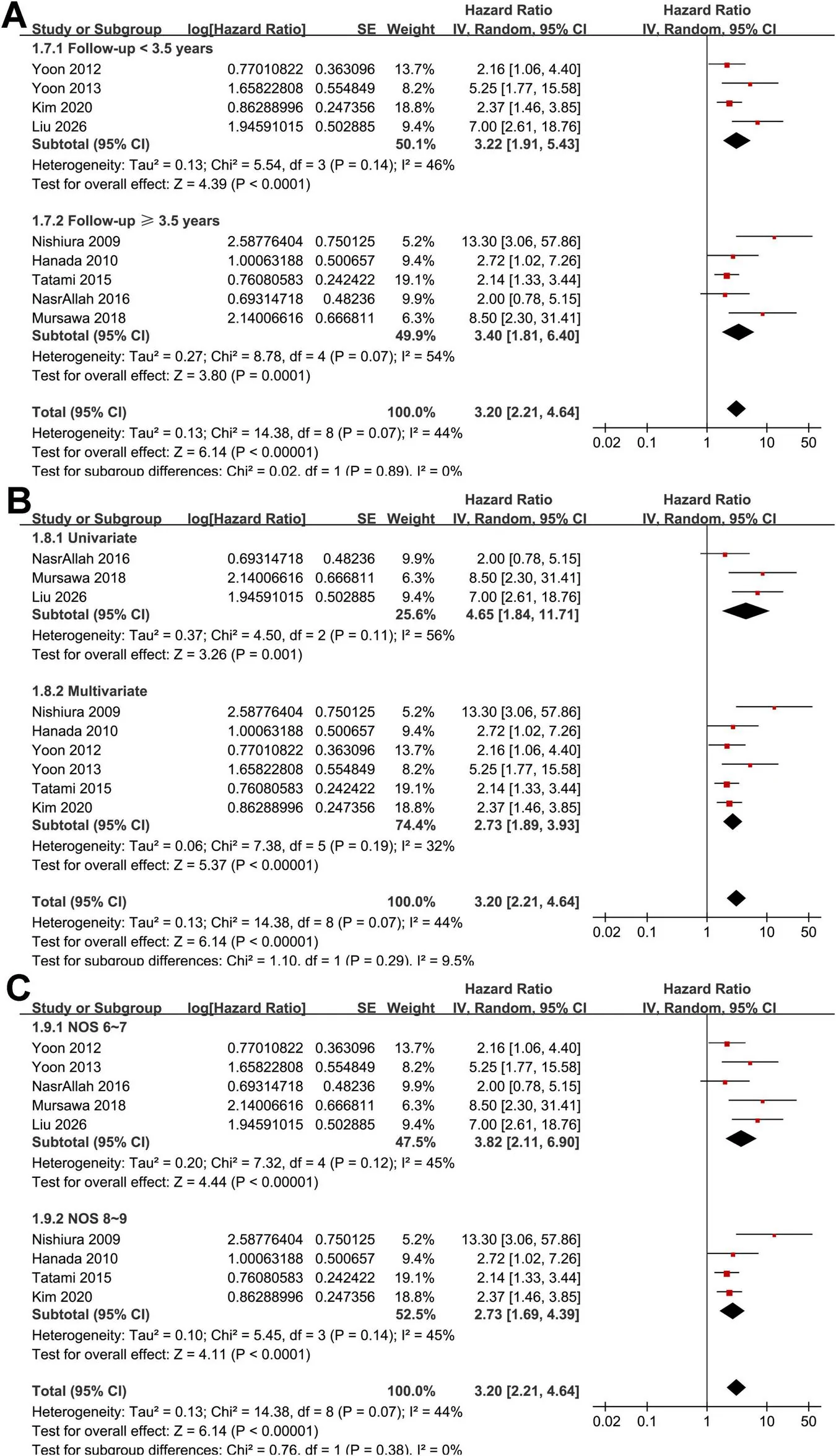
Forest plots showing the subgroup analyses of the association between ACI and CVEFS of patients with CKD: **(A)** forest plots for the subgroup analysis according to mean follow-up durations; **(B)** forest plots for the subgroup analysis according to analytic models; and **(C)** forest plots for the subgroup analysis according to study quality scores.

Further subgroup analyses showed consistent results in prospective and retrospective cohorts (HR: 3.44 vs. 3.11, *p* for subgroup difference = 0.81; Figure 2B), and in patients with pre-dialysis CKD, receiving dialysis, or kidney transplant recipients (HR: 3.14 vs. 2.97 and 8.50, *p* for subgroup difference = 0.33; Figure 2C). Further subgroup analysis suggested a stronger association between abdominal ACI and poor CVEFS in patients with mean age < 60 years, as compared to those ≥ 60 years (HR: 5.46 vs. 2.27, *p* for subgroup difference = 0.01; Figure 3A). The results were not statistically significant between studies with the proportion of men < 55% and those ≥ 55% (HR: 4.02 vs. 2.57, *p* for subgroup difference = 0.25; Figure 3B). Interestingly, a stronger association between abdominal ACI and poor CVEFS was observed in studies with the cutoff values of ACI ≥ 30% as compared to those < 30% (HR: 8.53 vs. 2.37, *p* for subgroup difference < 0.001; Figure 3C). Further subgroup analyses showed similar results in studies with the mean follow-up duration < 3.5 years and ≥ 3.5 years (HR: 3.22 vs. 3.40, *p* for subgroup difference = 0.89; Figure 4A), in studies reporting data from univariate and multivariate analyses (HR: 4.65 vs. 2.73, *p* for subgroup difference = 0.29; Figure 4B), and in studies with the NOS scores of 6∼7 and 8∼9 (HR: 3.82 vs. 2.73, *p* for subgroup difference = 0.38; Figure 4C).

Moreover, exploratory univariate meta-regression analysis showed that ACI cutoff value was the only study characteristic significantly associated with the strength of the association between ACI and CVEFS (coefficient = 0.033, *p* = 0.02; Table 3). Specifically, studies using higher ACI cutoff values tended to report stronger adverse associations between high ACI and poor CVEFS. No significant effects were observed for sample size, age, sex distribution, diabetes prevalence, follow-up duration, or study quality (all *p* > 0.05).

**TABLE 3:** Results of univariate meta-regression analysis for the association between ACI and CVEFS in patients with CKD.

Variables	HR for the association between ACI and CVEFS			
	Coefficient	95% CI	*p*-values	Adjusted R^2^
Sample size	−0.0012	−0.0038 to 0.0013	0.28	0%
Mean age (years)	−0.029	−0.083 to 0.024	0.24	39.3%
Men (%)	−0.030	−0.087 to 0.028	0.26	18.6%
DM (%)	−0.0048	−0.0335 to 0.0238	0.70	0%
Cutoff values of ACI (%)	0.033	0.006 to 0.060	0.02	100%
Follow-up duration (years)	0.037	−0.559 to 0.633	0.89	0%
NOS	−0.26	−0.63 to 0.11	0.14	32.5%

ACI, abdominal aortic calcification index; CI, confidence interval; CKD, chronic kidney disease; CVEFS, cardiovascular event-free survival; DM, diabetes mellitus; HR, hazard ratio; NOS, Newcastle–Ottawa Scale.

**FIGURE 5:**
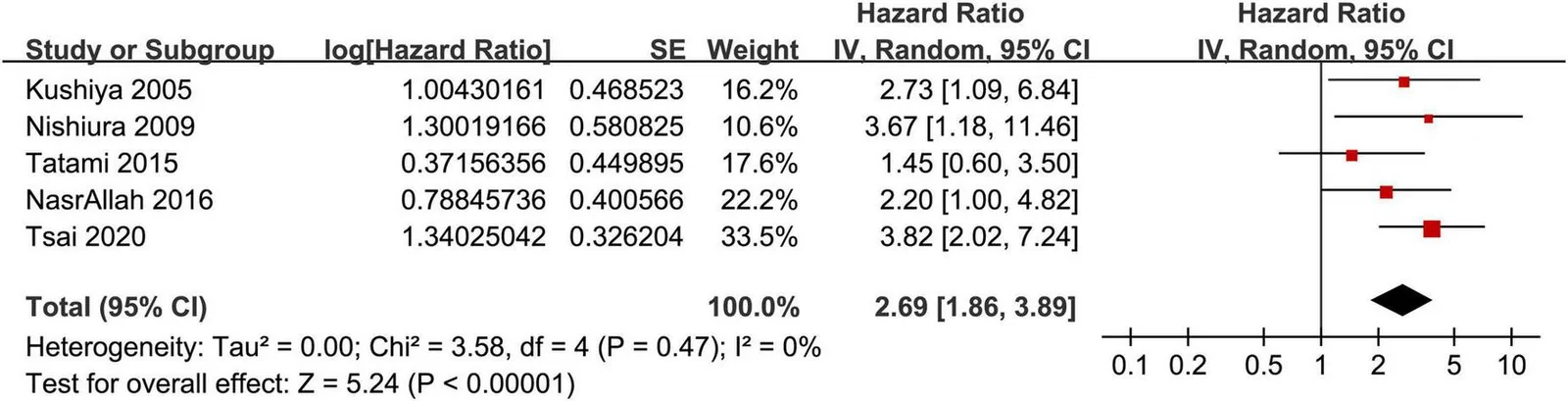
Forest plots showing the meta-analysis of the association between ACI and OS of patients with CKD.

Finally, the pooled results with five studies (19, 20, 24, 25, 28) suggested that a high ACI at baseline was also associated with poor OS in patients with CKD (HR: 2.69, 95% CI: 1.86 to 3.89, *p* < 0.001; Figure 5) with no significant heterogeneity (*p* for Cochrane Q test = 0.47; I^2^ = 0%). Similar findings were obtained using the REML random-effects model (HR: 2.68, 95% CI: 1.84 to 3.91; Supplementary Figure 1B), indicating that the results were robust to the choice of random-effects estimator. Sensitivity analyses by excluding one study at a time also showed similar results, with HR ranging from 2.25 to 3.07 (all *p* < 0.05), confirming the robustness of the overall estimate.

### Publication bias

**FIGURE 6:**
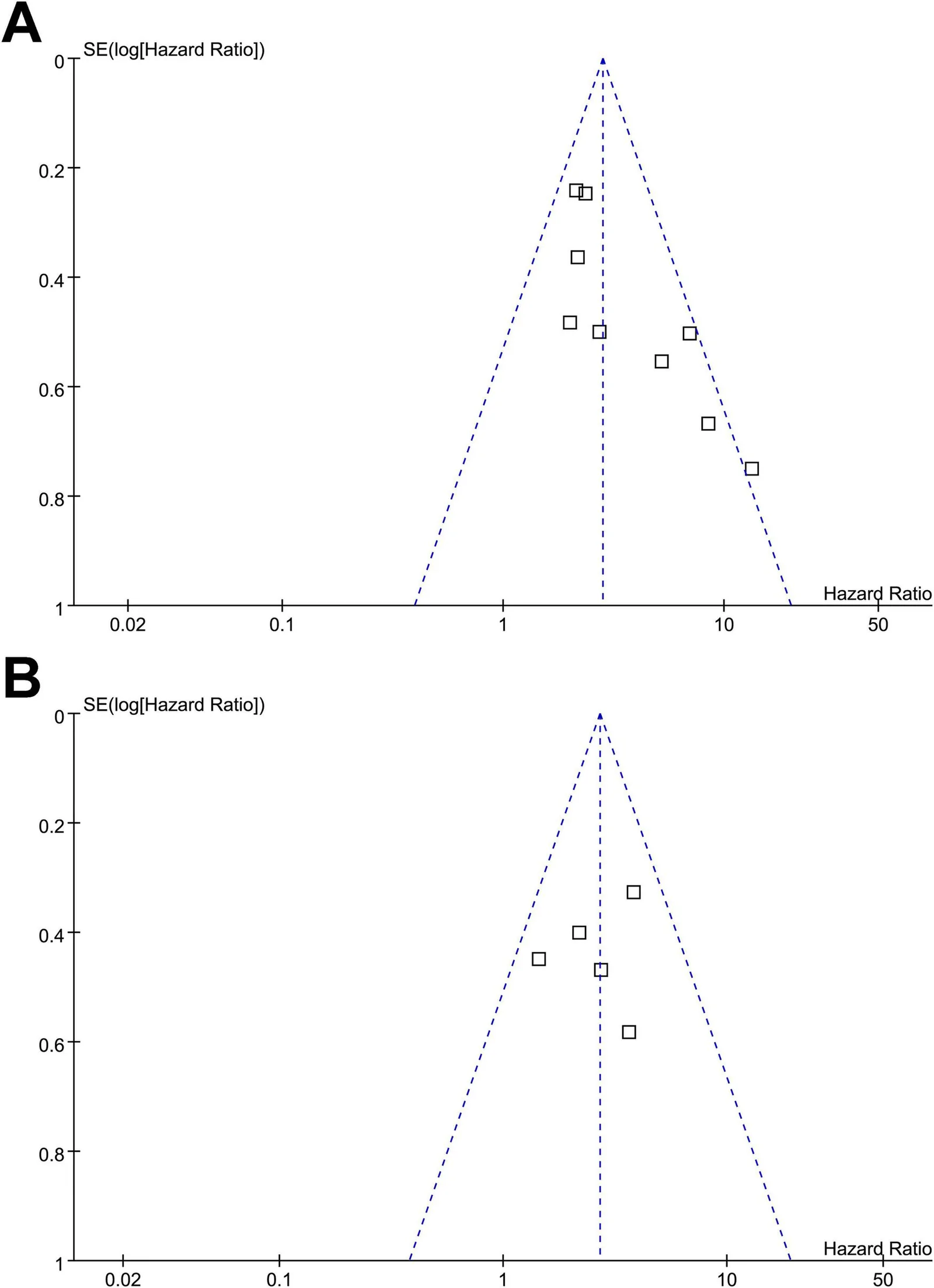
Funnel plots evaluating the publication bias for the meta-analyses: **(A)** funnel plots for the meta-analysis of the association between ACI and CVEFS of patients with CKD; and **(B)** funnel plots for the meta-analysis of the association between ACI and OS of patients with CKD.

Assessment of publication bias suggested no obvious evidence of reporting-related distortions. Funnel plots for CVEFS and OS (Figures 6A,B) appeared largely symmetrical, and Egger’s regression test for CVEFS was non-significant (*p* = 0.22), indicating no detectable small-study effects. However, these findings should be interpreted cautiously because only nine studies contributed to the CVEFS analysis, limiting the statistical power of publication bias assessments. Egger’s regression test was not performed for OS because only five studies were available, and therefore the possibility of publication bias for this outcome cannot be excluded.

## Discussion

The present meta-analysis systematically synthesized the available evidence regarding the prognostic significance of abdominal ACI in patients with CKD. By pooling data from 11 cohort studies involving a broad spectrum of CKD populations, including predialysis CKD, dialysis-dependent patients, and kidney transplant recipients, we found that a high baseline ACI was consistently associated with poorer CVEFS and OS. These findings were generally robust across sensitivity analyses and remained evident in most predefined subgroup analyses. Collectively, the results support the concept that AAC is not merely a marker of vascular aging but may also reflect a clinically meaningful burden of cardiovascular risk that carries important prognostic implications throughout the CKD continuum.

Several methods have been developed to assess AAC, including plain radiography-based semiquantitative scoring systems, ultrasonography, and CT-based approaches (36, 37). Compared with these methods, ACI offers several advantages. First, ACI is derived from CT images and quantitatively measures the proportion of the abdominal aortic wall affected by calcification, providing a continuous assessment of calcification burden rather than a categorical or semiquantitative estimate (38). Second, the CT-based protocol allows more objective and reproducible evaluation with reduced observer dependency (39). Third, ACI may be more sensitive to subtle differences in calcification severity and disease progression than traditional radiographic scores (25). These characteristics make ACI an attractive imaging biomarker for risk stratification and longitudinal assessment in CKD populations, in whom vascular calcification is highly prevalent and clinically relevant.

The observed association between elevated ACI and adverse outcomes is biologically plausible. Vascular calcification in CKD is a complex and actively regulated process driven by disturbances in calcium-phosphate metabolism, chronic inflammation, oxidative stress, accumulation of uremic toxins, and vascular smooth muscle cell osteogenic transformation (40, 41). Calcification of the abdominal aorta contributes to arterial stiffening, increased pulse-wave velocity, elevated systolic blood pressure, and widened pulse pressure, resulting in increased cardiac afterload and impaired coronary perfusion (42, 43). These hemodynamic alterations promote left ventricular hypertrophy, myocardial ischemia, and heart failure, thereby increasing the risk of cardiovascular events (42, 43). In addition, extensive vascular calcification may reflect advanced systemic atherosclerotic burden and generalized vascular injury, both of which are associated with increased mortality (44, 45). Therefore, ACI likely serves not only as a localized measure of abdominal aortic disease but also as a surrogate marker of cumulative cardiovascular damage and biological aging in patients with CKD (46).

Interestingly, subgroup analyses suggested a stronger association between ACI and CVEFS in studies with younger populations than in those with older populations. Several explanations may account for this finding. In younger patients, substantial AAC is relatively uncommon and may therefore represent a particularly severe vascular phenotype characterized by accelerated vascular aging and more profound metabolic abnormalities (47). Consequently, the prognostic implications of a high ACI may be more pronounced in younger individuals. In contrast, vascular calcification becomes increasingly prevalent with advancing age (48), potentially reducing its discriminatory value for risk prediction among older populations because calcification may reflect age-related changes in addition to disease-related pathology. Nevertheless, this finding should be interpreted cautiously because the subgroup analysis was based on a limited number of studies and study-level rather than patient-level data, making it susceptible to ecological bias, residual confounding, and limited statistical power. Furthermore, as multiple subgroup analyses were performed, the observed association should be considered exploratory and hypothesis-generating rather than confirmatory.

Another notable finding was the stronger association between ACI and CVEFS in studies using higher ACI cutoff values. This observation was further supported by the meta-regression analysis, which identified ACI cutoff value as the only significant study-level characteristic associated with the strength of the observed effect. One possible explanation is that higher cutoff values identify patients with more advanced and clinically significant vascular calcification, thereby enriching the high-ACI group with individuals at particularly elevated cardiovascular risk, whereas lower thresholds may classify patients with relatively mild calcification as high-risk, reducing the prognostic contrast between groups. However, these findings should be interpreted cautiously because the included studies used heterogeneous methods to define high ACI, including predefined thresholds, median values, tertiles, and ROC-derived cutoffs, resulting in substantial variability in the cutoff values. Therefore, the observed differences likely reflect study-level variation in exposure definition rather than the identification of an optimal or standardized prognostic threshold for ACI. From a clinical perspective, the present findings support the use of ACI as a quantitative indicator of vascular calcification burden associated with prognosis, rather than endorsing any specific cutoff value for risk stratification. Until standardized and externally validated thresholds become available, ACI should be interpreted in conjunction with the overall clinical context and established cardiovascular risk factors, rather than being used in isolation to guide clinical decision-making. Future large-scale prospective studies and individual participant data meta-analyses using standardized ACI assessment protocols are warranted to establish clinically applicable cutoff values and determine their incremental prognostic value.

The strengths of this meta-analysis should be acknowledged. To our knowledge, this is the first systematic review and meta-analysis specifically evaluating the prognostic significance of ACI in patients with CKD. The literature search was comprehensive and up to date, covering multiple international and Chinese databases. Only longitudinal cohort studies were included, strengthening the temporal relationship between baseline ACI and subsequent outcomes. Furthermore, we performed extensive sensitivity analyses, prespecified subgroup analyses, and meta-regression analyses to explore the robustness of the findings and potential sources of heterogeneity. The consistency of the results across most subgroup analyses and the stability of the pooled estimates after sequential study exclusion further support the reliability of the conclusions.

However, several limitations should also be considered. First, although most studies were prospective, several retrospective cohorts were included, which may be susceptible to selection bias and incomplete data collection. Second, the included studies were conducted almost exclusively in East Asia, with only one study from Egypt and no studies from Western populations. Consequently, the generalizability of the present findings to other ethnic groups and healthcare settings remains uncertain because the prevalence, progression, and clinical implications of vascular calcification may differ according to genetic background, environmental exposures, healthcare systems, and clinical practice patterns. In addition, considerable clinical and methodological heterogeneity existed among the included studies. The study populations included patients across different stages of CKD, including pre-dialysis CKD, dialysis-dependent CKD, and kidney transplant recipients. Although subgroup analyses according to CKD stage did not demonstrate significant differences, the kidney transplant subgroup was represented by only one study. Consequently, the prognostic value of ACI in kidney transplant recipients remains uncertain and should be interpreted cautiously until confirmed by additional studies. In addition, the methods used to define high ACI and the definitions of cardiovascular events varied across studies, limiting the direct comparability and clinical interpretability of the pooled CVEFS outcome. Although subgroup and meta-regression analyses were performed to explore potential sources of heterogeneity, these analyses were based on study-level data and should be considered exploratory. Therefore, the findings should be interpreted cautiously. Third, although adjusted estimates from the most comprehensively adjusted models were preferentially extracted whenever available, four included studies reported only unadjusted HRs and therefore could not account for potential confounding. Consequently, the pooled estimates were derived from a combination of adjusted and unadjusted effect estimates, which may have influenced the magnitude of the observed associations. Although subgroup analysis showed no statistically significant difference between studies reporting adjusted and unadjusted HRs, this finding should not be interpreted as evidence that confounding was absent because the analysis was based on a limited number of studies and had limited statistical power. In addition, adjustment strategies varied considerably across studies and residual confounding from unmeasured factors—including inflammatory status, mineral metabolism abnormalities, medication use, nutritional status, and cardiovascular comorbidities—cannot be excluded. Therefore, the observed associations should be interpreted cautiously and should not be regarded as establishing an independent prognostic effect of ACI. Fourth, publication bias could not be reliably excluded because the number of available studies was limited, particularly for OS. Fifth, as with all meta-analyses of observational studies, the present findings demonstrate associations rather than causality. Therefore, it cannot be concluded that reducing ACI would necessarily improve clinical outcomes. Finally, the subgroup and meta-regression analyses were based on a relatively small number of studies and study-level characteristics. Therefore, these analyses should be considered exploratory and interpreted cautiously because they may be affected by ecological bias, limited statistical power, and multiple comparisons.

The present findings may have important clinical implications. Because ACI can be quantified objectively using CT imaging and reflects cumulative vascular calcification burden, it may provide useful prognostic information for identifying CKD patients at elevated cardiovascular risk. However, the current evidence demonstrates an association between higher ACI and adverse outcomes rather than establishing that ACI provides incremental prognostic value beyond established clinical risk factors or existing risk prediction models. Accordingly, ACI should currently be considered a potential prognostic marker rather than a therapeutic target or a validated tool for routine risk stratification. Future large-scale prospective studies should establish standardized protocols for ACI measurement, determine clinically meaningful cutoff values, evaluate whether ACI improves risk prediction beyond routine clinical predictors using appropriate measures of predictive performance, and clarify whether changes in ACI over time are associated with subsequent clinical outcomes. Individual participant data meta-analyses may also help identify patient subgroups that derive the greatest prognostic benefit from ACI assessment.

## Conclusion

In conclusion, this meta-analysis suggests that a high ACI is associated with poorer CVEFS and OS in patients with CKD. These findings support the potential prognostic relevance of ACI as an imaging biomarker in this population. However, whether ACI provides incremental prognostic value beyond established clinical risk factors or existing risk prediction models remains to be established. The findings should therefore be interpreted cautiously given the observational nature of the included studies and the clinical and methodological heterogeneity across studies. Further large-scale prospective studies are warranted to confirm these findings, establish standardized ACI assessment protocols and clinically meaningful cutoff values, and determine the incremental prognostic utility of ACI for clinical risk stratification.

## Data Availability

The original contributions presented in this study are included in this article/Supplementary material, further inquiries can be directed to the corresponding author.
