# Supplementary material for: Abdominal aortic calcification index and prognosis in patients with chronic kidney disease: a meta-analysis
Source: Front Med (Lausanne). 2026 Sep 9;13:1907104. doi: 10.3389/fmed.2026.1907104 (PMC13597373; doi:10.3389/fmed.2026.1907104)
Supplement: Supplementary file 1 [file Table_1.docx]

**Detailed search strategy for each database**

**PubMed**

("aortic calcification index"[Title/Abstract] OR "abdominal aortic calcification index"[Title/Abstract] OR "abdominal aortic calcification percentage"[Title/Abstract] OR "aortic calcification percentage"[Title/Abstract] OR ACI[Title/Abstract]) AND ("Renal Insufficiency, Chronic"[Mesh] OR "Kidney Failure, Chronic"[Mesh] OR "Renal Dialysis"[Mesh] OR "Kidney Transplantation"[Mesh] OR renal[Title/Abstract] OR kidney[Title/Abstract] OR "chronic kidney disease"[Title/Abstract] OR CKD[Title/Abstract] OR "end-stage renal disease"[Title/Abstract] OR ESRD[Title/Abstract] OR dialysis[Title/Abstract] OR hemodialysis[Title/Abstract] OR haemodialysis[Title/Abstract] OR "peritoneal dialysis"[Title/Abstract]) AND (mortality[Title/Abstract] OR death[Title/Abstract] OR survival[Title/Abstract] OR prognosis[Title/Abstract] OR prognostic[Title/Abstract] OR outcome*[Title/Abstract] OR event*[Title/Abstract] OR "cardiovascular event*"[Title/Abstract] OR "major adverse cardiovascular event*"[Title/Abstract] OR MACE[Title/Abstract])

**Embase**

('aortic calcification index':ti,ab OR 'abdominal aortic calcification index':ti,ab OR 'abdominal aortic calcification percentage':ti,ab OR 'aortic calcification percentage':ti,ab OR ACI:ti,ab) AND (exp chronic kidney failure/ OR exp chronic kidney disease/ OR exp hemodialysis/ OR exp dialysis/ OR exp renal replacement therapy/ OR renal:ti,ab OR kidney:ti,ab OR 'chronic kidney disease':ti,ab OR CKD:ti,ab OR ESRD:ti,ab OR dialysis:ti,ab OR hemodialysis:ti,ab OR haemodialysis:ti,ab) AND (exp mortality/ OR exp survival/ OR exp prognosis/ OR mortality:ti,ab OR death:ti,ab OR survival:ti,ab OR prognosis:ti,ab OR prognostic:ti,ab OR outcome*:ti,ab OR event*:ti,ab OR 'cardiovascular event*':ti,ab OR MACE:ti,ab)

**Web of Science**

TS=(("aortic calcification index" OR "abdominal aortic calcification index" OR "abdominal aortic calcification percentage" OR "aortic calcification percentage" OR ACI) AND (renal OR kidney OR "chronic kidney disease" OR CKD OR ESRD OR dialysis OR hemodialysis OR haemodialysis OR "peritoneal dialysis") AND (mortality OR death OR survival OR prognosis OR prognostic OR outcome* OR event* OR "cardiovascular event*" OR MACE))

**Wanfang**

("主动脉钙化指数" OR "腹主动脉钙化指数" OR "腹主动脉钙化率" OR "主动脉钙化率" OR ACI) AND (肾病 OR 慢性肾脏病 OR 慢性肾病 OR CKD OR 终末期肾病 OR ESRD OR 透析 OR 血液透析 OR 腹膜透析) AND (死亡 OR 死亡率 OR 生存 OR 预后 OR 结局 OR 心血管事件 OR 不良心血管事件)

**CNKI**

("主动脉钙化指数" OR "腹主动脉钙化指数" OR "腹主动脉钙化率" OR "主动脉钙化率" OR ACI) AND ("慢性肾脏病" OR "慢性肾病" OR CKD OR "终末期肾病" OR ESRD OR "血液透析" OR "腹膜透析" OR 透析) AND (死亡 OR 死亡率OR 生存OR 预后OR 结局OR 心血管事件OR 主要不良心血管事件)
