# Supplementary material for: Abdominal aortic calcification index and prognosis in patients with chronic kidney disease: a meta-analysis
Source: Front Med (Lausanne). 2026 Sep 9;13:1907104. doi: 10.3389/fmed.2026.1907104 (PMC13597373; doi:10.3389/fmed.2026.1907104)
Supplement: Supplementary file 2 [file Table_2.docx]

**Supplemental Table 1** Definition of cardiovascular events in each study reporting the outcome of CVEFS

| Study | Definition of cardiovascular events |
| --- | --- |
| Nishiura 2009 | Ischemic heart disease, chronic heart failure, dissecting aortic aneurysm, cerebral infarction, cerebral bleeding, or sudden death. |
| Hanada 2010 | Cardiovascular events in pre-dialysis CKD; reported events included ischemic heart disease and congestive heart failure. |
| Yoon 2012 | Nonfatal CV events included coronary heart disease, cerebrovascular disease, peripheral/pulmonary vascular disease, and heart failure. |
| Yoon 2013 | CV events included coronary heart disease, cerebrovascular disease, peripheral/pulmonary vascular disease, and heart failure. |
| Tatami 2015 | CV death, nonfatal stroke, nonfatal myocardial infarction, angina pectoris requiring revascularization, and heart failure requiring admission. |
| NasrAllah 2016 | New major cardiovascular events during follow-up; the article did not provide a more detailed component-level definition in the available text. |
| Mursawa 2018 | Post-transplant cardiovascular events; reported events included angina pectoris and atrial fibrillation. |
| Kim 2020 | Cardiovascular events included coronary heart disease, heart failure, cerebrovascular disease, peripheral vascular disease, or pulmonary vascular disease. |
| Liu 2026 | Myocardial infarction, acute or chronic heart failure, arrhythmia, cerebrovascular accident, coronary artery disease, etc. |

CKD, chronic kidney disease; CV, cardiovascular; CVEFS, cardiovascular event-free survival;
