# Supplementary material for: Abdominal aortic calcification index and prognosis in patients with chronic kidney disease: a meta-analysis
Source: Front Med (Lausanne). 2026 Sep 9;13:1907104. doi: 10.3389/fmed.2026.1907104 (PMC13597373; doi:10.3389/fmed.2026.1907104)
Supplement: Supplementary file 3 [file Table_3.docx]

**Supplemental Figure 1** Sensitivity analyses for the outcome of CVEFS and OS with REML random-effects model; A, Forest plots showing the meta-analysis of the association between ACI and CVEFS of patients with CKD with REML random-effects model; and B, Forest plots showing the meta-analysis of the association between ACI and OS of patients with CKD with REML random-effects model;
